# Efficient Data Gathering in 3D Linear Underwater Wireless Sensor Networks Using Sink Mobility

**DOI:** 10.3390/s16030404

**Published:** 2016-03-19

**Authors:** Mariam Akbar, Nadeem Javaid, Ayesha Hussain Khan, Muhammad Imran, Muhammad Shoaib, Athanasios Vasilakos

**Affiliations:** 1 COMSATS Institute of Information Technology, Islamabad 44000, Pakistan; mariam.akbar@gmail.com (M.A.); ayeshahussainkhan6@gmail.com (A.H.K.); 2 College of Computer and Information Sciences, King Saud University, Riyadh 11451, Saudi Arabia; cimran@ksu.edu.sa (M.I.); muhshoaib@ksu.edu.sa (M.S.); 3 Lulea University of Technology, Lulea 97187, Sweden; vasilako@ath.forthnet.gr

**Keywords:** energy consumption, underwater wireless sensor network, direct transmission, mobile sink, courier nodes

## Abstract

Due to the unpleasant and unpredictable underwater environment, designing an energy-efficient routing protocol for underwater wireless sensor networks (UWSNs) demands more accuracy and extra computations. In the proposed scheme, we introduce a mobile sink (MS), *i.e.*, an autonomous underwater vehicle (AUV), and also courier nodes (CNs), to minimize the energy consumption of nodes. MS and CNs stop at specific stops for data gathering; later on, CNs forward the received data to the MS for further transmission. By the mobility of CNs and MS, the overall energy consumption of nodes is minimized. We perform simulations to investigate the performance of the proposed scheme and compare it to preexisting techniques. Simulation results are compared in terms of network lifetime, throughput, path loss, transmission loss and packet drop ratio. The results show that the proposed technique performs better in terms of network lifetime, throughput, path loss and scalability.

## 1. Introduction

Presently, we are in the modern era of information, and almost every field is equipped with technology to stay updated. Underwater wireless sensor networks (UWSNs) are becoming popular due to their advantages, like pollution monitoring, oil extraction monitoring and aquiculture monitoring [1]. These networks consist of a number of wireless sensors (nodes) for specific tasks, such as sensing the physical attributes of the water environment. After sensing the desired information, nodes forward it to the sink. This transmission can be direct or multi-hop, where nodes relay the data of other nodes. In water, acoustic signals are used for communication; these cause a high delay in communication because of their speed (1500 m/s). The reason is radio or optical signals are affected by a huge amount of scattering and absorption loss. Unlike terrestrial WSNs, acoustic channels have high energy consumption, limited bandwidth and low transmission speed, as shown in Table 1 [2]. These nodes can bear harsh weather conditions in deep sea and are expected to stay alive on batteries without getting recharged. However, these operate on limited battery power, and it is almost impossible to recharge or replace the batteries.

Commonly, sea areas that need to be sensed are large and deep. The underwater deployed nodes have complex transceivers. To limit the interference, nodes are forced to communicate at short distances. When the sink is far away, then multi-hop communication schemes are used. The nodes that relay the data of other nodes consume more energy. If a node continuously participates in relaying data of other nodes, it will exhaust its energy, get disconnected from the network and leave the information or an energy hole. Network lifetime (NLT) depends on the lifetime of nodes deployed in the field. To enhance the NLT, efficient management of energy consumption is needed.

In UWSNs, different approaches are used for data collection: the autonomous underwater vehicle (AUV) (also named the mobile sink (MS); it can get refueled or recharged periodically; because of this reason, it is assumed that the MS has no constraint with respect to energy) -based schemes use direct or multi-hop communication between the sensor node and the sink [3]; courier node (CN) (these are nodes that are equipped with extra resources) -based schemes for data gathering [4]; and clustering-based schemes to relay the data of member nodes (MNs) [5]. In the first approach, the AUV moves in the field and gathers data from the nodes through multi-hop or direct communication. In the second approach, the CN moves on predefined paths in the network and receives data from nodes at a minimum distance or far away nodes with multi-hop transmission. Like MS, CN also has no constraint with respect to energy consumption. The optimal number of CNs is deployed in the network; otherwise, the cost of the network may increase. In the latter one, a network is logically partitioned into clusters, such that each cluster selects a CH depending on the defined criteria. In [5], after the selection of the CH, CHs further divide clusters into sub-clusters and select a delegate node (path node (PN)) for data gathering. Nodes in the sub-cluster forward their data through the PN, and the PN forwards these data to the sink.

Each of the above schemes has advantages and disadvantages. Multi-hop communication minimizes the energy consumption of far away nodes, while nodes that rely on the data of other nodes consume more energy. Furthermore, clustering schemes minimize the energy consumption of MNs; however, CHs have a greater burden. To minimize energy consumption, we propose a three-dimensional sink mobility (3D-SM) scheme. We consider a three-dimensional rectangular network; for simplicity, the network volume is divided into four sub-regions, each called a rectangular cuboid (RC). In one of the RCs, an MS is deployed that gathers data from the nodes present there. The remaining RCs are equipped with CNs that move in, to gather data from the nodes. The CNs send the received data to the MS at a minimum distance. All of the transmissions made by the nodes are directed to the MS or CNs. In this way, the energy consumption of the network is minimized, such that the NLT is prolonged.

The rest of the paper is organized as follows. In Section 2, related work is presented. In Section 3, the motivation and contribution are given. In Section 4, the framework and system description are given. In Section 5, simulation results are presented. In Section 6, scalability analysis is given. In Section 7, the fixed routing and dynamic sink mobility are given. After that, in Section 8, a discussion about the performance trade-offs is given. Finally, Section 9 concludes this paper.

## 2. Related Work

In this section, an overview of existing techniques is given. The types of routing schemes used in UWSNs are depth-based routing, probabilistic routing, data gathering schemes using AUVs, *etc*. Many techniques introduced CNs in depth-based routing to minimize the load on forwarding nodes. Wahid and Dongkyun in [6] investigate UWSN localization-free routing schemes that minimize energy consumption. In this scheme, nodes at a high depth forward their data to nodes that are at a low depth. During the data forwarding process, it considers the residual energy of the node as a routing metric. Nodes with a high depth only forward their data to low depth nodes that have residual energy greater than a threshold value.

Javaid *et al.* [7] proposed depth-based routing schemes; they empowered them through a delay-efficient priority factor and holding time (also delay sensitive). This scheme targets minimizing end-to-end delay at the cost of the decreased throughput of the network. They addressed delay sensitivity through choosing optimal data forwarders in a low depth region. Furthermore, to avoid the energy holes and data loss, they selected those nodes as forwarders that had a high number of neighboring nodes. They also introduced the adaptive mobility of CNs. This scheme is localization free.

The authors proposed a directional flooding-based routing protocol in [8]. This scheme checks the link quality of nodes that are participating in the flooding. The number of nodes participating in flooding is controlled to avoid packet flooding in the whole field. If there are flooding nodes with poor link quality, then directional flooding-based routing (DFR) allows more nodes to forward data. This is done for achieving reliable data delivery. On the other hand, a few nodes are enough to forward the data.

The authors proposed a scheme for an AUV-aided underwater routing protocol (AURP) in [3]; it uses the controlled mobility of multiple AUVs, as well as heterogeneous acoustic communication channels. The role of relay nodes is replaced by AUVs; they collect data from gateway nodes and send these to the sink. In this way, the data transmissions are minimized. AURP used the controlled mobility of AUVs, which results in short-range high data rates. By simulation, AURP shows an improved delivery ratio and minimizes the energy consumption of nodes.

Mobicast [9] addressed the energy problem in the underwater environment for achieving maximum throughput. Mobicast considered a network that is divided into three-dimensional zones. Data gathering paths are predefined; the AUV moves in the zones for receiving data from the nodes. There are two phases of the protocol: the first is data collection within the three-dimensional zone; the second phase is awaking nodes in the next zone where the AUV is heading for data collection. When the AUV enters the next three-dimensional zone, the sensors go into active mode and deliver the sensed data. Due to the division of the field into different zones and introducing sleep/awake modes, this protocol minimizes the energy consumption of the nodes. This leads to the maximized throughput of the system.

Underwater deployed nodes have movement due to water currents, which affect the energy efficiency of the network. In the underwater environment, the water medium causes long propagation delays; to avoid these, multi-hop transmission is preferred. While relaying the data of far away nodes, the nodes closer to the sink have a greater burden and dissipate energy early; however, the overall energy consumption is minimized. In the adaptive power controlled routing protocol (APCR), in [10], the authors jointly addressed the mobility of both the surface sink and nodes. Furthermore, the coverage is through the power control capabilities. The authors presented a scheme of an adaptive sink redeployment that reduces the total energy consumption in the APCR. This strategy does not compromise the average end-to-end delay and delivery ratio.

In [11], the authors analyzed a heterogeneous underwater network. Nodes have been assigned different categories based on their functionalities. The head node is selected, and it is responsible for gathering data from the neighboring nodes. Head nodes are distributed over the network, and they collect the data from respective neighboring nodes and forward the data to an AUV, which is taking a data-gathering tour. The selection criteria of the head node and its placement are not discussed, which leads to non-uniform energy consumption. The rapid depletion of energy leads to decreased NLT.

In [5], the authors used an AUV working as an MS. It effectively saves energy by reducing the transmission range of nodes. The tour points are predefined for data gathering from the network. Due to drastic environmental conditions, nodes are mobile and randomly changing their positions; therefore, the authors called them the probabilistic neighborhood of the AUV stop. The AUV finds these probabilistic neighborhoods of its tour stops for a specific time period, and this is called the probe interval. In the next step, the AUV identifies the nodes and creates a communication schedule for these nodes. The AUV waits at each stop until all data are collected from the neighbors. As the neighbors are changing due to the probabilistic nature, nodes consume non-uniform energy, and some nodes deplete more quickly; also, the end-to-end delay of the scheme increases.

Domingo *et al.* [12] presented an analysis of energy consumption in shallow and deep water. Moreover, they applied three different communication types to analyze energy consumption. These three types are: relaying, direct transmission and clustering. The results show that direct communication in the underwater environment performs poorly, because when the distance increases between the node and sink, the acoustic interference causes packet drops. This results in the low throughput of the network. The relaying scheme performs well in deep water, as it minimizes the overall network energy consumption; however, it increases the network complexity. The third type is clustering, in which the network is partitioned into a number of clusters, and each cluster selects a CH for data gathering from the MNs. After receiving data from MNs, the CH forwards them to the sink.

A comprehensive overview of the discussed protocols is given in Table 2 for better understanding.

### 2.1. Counterpart Schemes in Brief

This section gives a brief discussion of the schemes compared to our protocol.

#### 2.1.1. AUV Visits Path Nodes

The designed UWSN is three-dimensional, where the nodes’ depth is the same by anchoring them to the ocean floor. The AUV is also moving at a constant depth, *i.e.*, $D_{avu}<D$. In this way, the authors simplified the problem, and they simulated the two-dimensional field, with a random uniform node distribution at a fixed depth *D*. This two-dimensional Plane A is divided into several sub-regions, which are named clusters. Initially, when the network starts, the AUV partitions the network into clusters through a Voronoi generator point and broadcasts the cluster information. Nodes use this information to identify the cluster to which they belong. After the cluster and node association phase, MNs select a delegate node known as the CH. The selected CH in each cluster further divides the cluster into several sub-clusters. In each sub-cluster, a PN is selected through the CH, which gathers primary data from MNs and relays them to the AUV. The CH broadcasts the PN information throughout the cluster. After this phase, the AUV takes another tour, which is called the data gathering tour; it visits each PN for which the CH informs of its address.

Data gathering is completed in three steps: MNs send their sensed data to the PN; in the next step, PNs send the received data to the AUV; and in the final step, the AUV sends all of the received data from the PNs to the sink. PNs gather data continuously, except the interval in which they transfer their data to the AUV. At the start of data gathering tour, the AUV visits each cluster and gets the list of PNs in that cluster. After obtaining the lists from each cluster, the AUV visits each PN in the order of the list. After completing one tour, the AUV returns to the start point and sends data to the surface sink. TDMA is used for multiplexing the link between the MNs and PN. The PN assigns the time slots to the MNs for data sending. To avoid the interference between the clusters, CDMA is employed. CHs with the AUV and the PN with the CH only exchange the control information, and they share the contention-based control channel of $F_{control}$. Between the AUV and sink, CDMA with dedicated orthogonal code is employed.

#### 2.1.2. Data-Driven Routing Protocol

The data-driven routing protocol (DDRP) has static nodes, and the MS can move in the network. The deployed nodes and MS have the same communication range. Two nodes can communicate if they are within the communication range. The MS has unlimited energy, while the nodes are equipped with limited energy. The MS broadcasts a control packet, and the nodes that receive that packet set themselves as one-hop neighbors. This control packet contains the MS identification, time stamp (when the control packet is issued) and the control interval, which can be affected by the speed of the MS or the communication range. The nodes that receive this control packet do not further broadcast it. The data packets contain information about the distance from the MS, and it records the shortest distance for data sending. If the value of Dist2mSink=2, this means that the node is two hops away from the MS. The maximum allowed value of Dist2mSink=K, where, K≥1. If the value of Dist2mSink is greater than K+1, this means that the node has no route to the MS. Further, the deployed nodes are divided into three types on the basis of their distance from the sink:

- The nodes that are present in the communication range of the MS send their data directly, and they are one-hop neighbors (O-nodes).
- The nodes sharing the communication range and that have a valid path to the MS are called M-nodes.
- The nodes that do not have a valid route to the MS and that have infinite hops are called I-nodes.

This protocol has the following working principle: Initially, at the start of the network, each node is an I-node; because at that time, the MS has not started gathering data and none of the nodes have received any control packet. As the network evolves, the MS starts it tour, and nodes receive control packets and set themselves as O-nodes in its path. When a potential two-hop neighbor overhears a data transmission made by an O-node, it updates its routing table. When this two-hop neighbor sends its data packet, it sets its Dist2mSink as two and also includes it in the data packet sent. In this way, the M-nodes set their routing table for data delivery. The authors give a brief analysis of the proposed scheme by varying the number of MSs in the same network area. To perform a fair analysis, we selected the case of four MSs.

## 3. Motivation and Contributions

**Figure 1:**
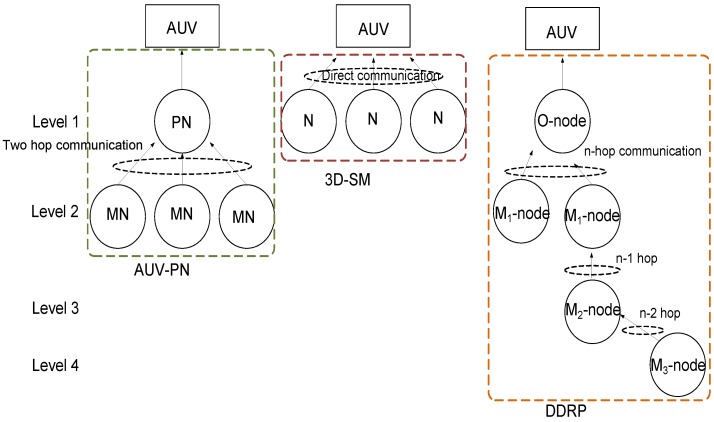
Comparison of the proposed and the counter schemes.

Designing an underwater network architecture is a hard task due to the drastic underwater conditions. The communication model used underwater is different compared to the TWSN as given in Table 1. We proposed a scheme, 3D-SM, which has better NLT and throughput. Nodes deployed in the field transmit their sensed data to the MS as these come in its transmission range. We compare it to the existing protocol DDRP [13], which is two-dimensional with the radio model for transmissions. For a fair comparison, we convert DDRP into a three-dimensional underwater environment and applied the acoustic model for transmissions. The mobility pattern of the MS is random in DDRP, and there is no restriction that it visits all of the regions for data gathering. Due to the random mobility, it may visit a single region more than once while leaving other regions unattended, because the round trip time and number of visits are fixed. In AUV-PN [5], the AUV visits pre-identified locations for gathering data. The AUV travels in the network where nodes are deployed, partitions the network into clusters and provides the partition information to nodes through the control packet. Nodes in each cluster select the CH, then the CH further divides the cluster into sub-clusters. The CH nominates a PN for each sub-cluster for receiving data from the MNs. The AUV then starts its tour to receive data and acquires the list of PNs from the CHs to collect data. In Figure 1, a comparison of three schemes (two existing and one proposed) is given. From the figure, it is obvious that existing schemes have multi-hoping and route finding, which consume energy.

In these routing protocols, DDRP has a coverage problem: all of the nodes are not able to send their data to the MS, as it has a random trajectory. Furthermore, it is using direct communication, and the nodes have to wait for their turn. AUV-PN has sink mobility, as well as clustering. Nodes have limited battery power, and for prolonging the network lifetime, balanced energy consumption is required. Nodes consume energy in cluster formation and PN selection. Furthermore, when the PN forwards the data gathered from the MN, it consumes more energy. This leads to quick energy depletion.

We have addressed the following challenges in underwater acoustic networks.

- Network energy consumption minimization.
- End-to-end delay minimization.

In the proposed scheme, nodes directly transmit data to the MS and do not relay the data of far away nodes. The scheme is designed in such a way that each node gets a chance to deliver the sensed data. In this way, nodes save their energy, which leads to prolonged NLT. To cope with the limitations of underwater transmissions, the MS gathers data from the RC where it is deployed. CNs collect data from the remaining RCs that are not considered to be in the trajectory of the MS.

**Figure 2:**
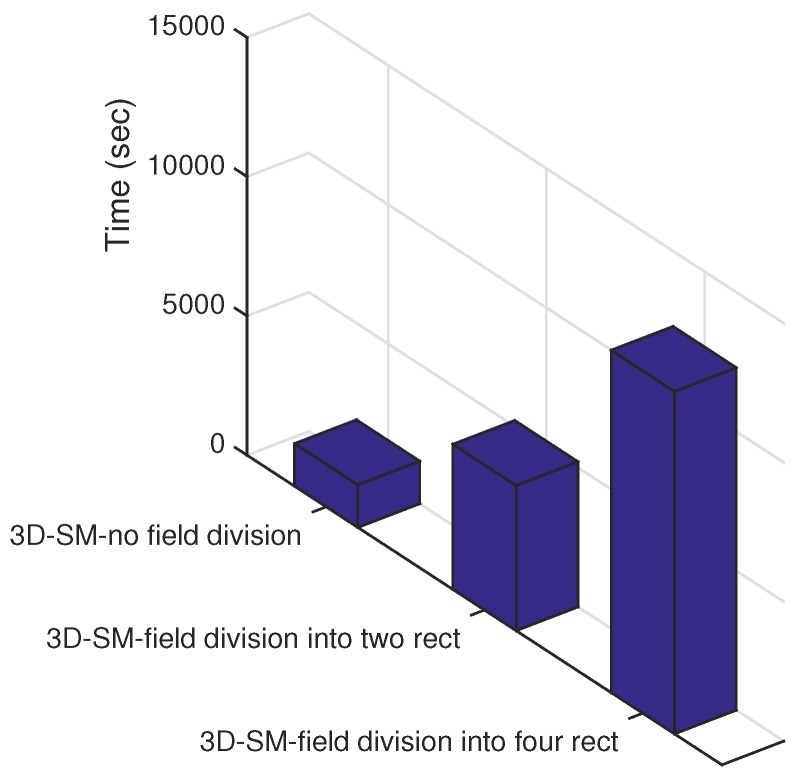
Impact of sub-regions on NLT.

Let us consider three different cases: (i) the network field is not logically divided; (ii) the network field is divided into two logical sub-regions; and (iii) the network field is logically divided into four sub-regions. The trajectories of the MS(s)/CN(s) are kept the same for all of the three cases. As the next step, we test the three mentioned cases via simulations; results are shown in Figure 2. From this figure, it is clear that the network lifetime increases as the number of logically-divided sub-areas is increased. However, the installation cost of the MS/CN prohibits further division (one AUV costs approximately 50,000 USD [14]). From our discussion till now, the solution seems to be static. Rather than static, our solution is adaptive, *i.e.*, we use the following formula for finding the number of region “RCs”.

$$RCs=\lceil \frac{x+y+x}{2n}\rceil$$

where *x*, *y* and *z* are the corresponding network field coordinates and *n* is the number of nodes.

## 4. Framework and Formal Definition of the Problem

Here, we describe the nodes’ relation with the MS or the CNs’ relation. We will also explain the network model.

### 4.1. Relationship between Nodes and MS

**Figure 3:**
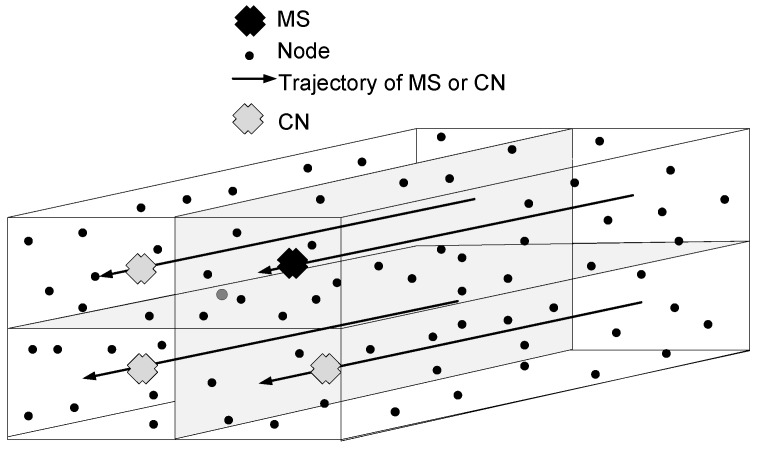
Network model.

**Figure 4:**
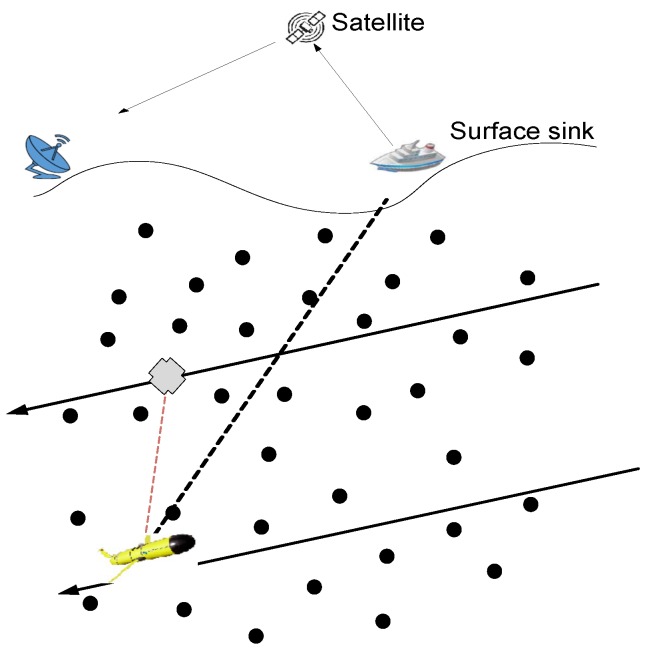
Courier node (CN) to mobile sink (MS) transmission.

UWSNs can be represented by a directed graph G=(V,E), where *V* is the set of vertices (nodes), *i.e.*, ∣V∣=n, where, n= 1, 2, 3, ..., 300. *E* are the edges that are links between the nodes. We consider the cuboid field of dimensions 500 m × 500 m × 1000 m, which is further logically divided into four RCs, shown in Figure 3. In one RC, the MS gathers the data from the nodes, and in the remaining three RCs, CNs are appointed to collect sensed data from the nodes. CNs further forward the received data to the MS, shown in Figure 4. The MS and CNs stop at locations defined on their trajectories. The set of stop locations of the MS and CNs is defined as $S=s_{1},s_{2},...,s_{m}$.

**Figure 5:**
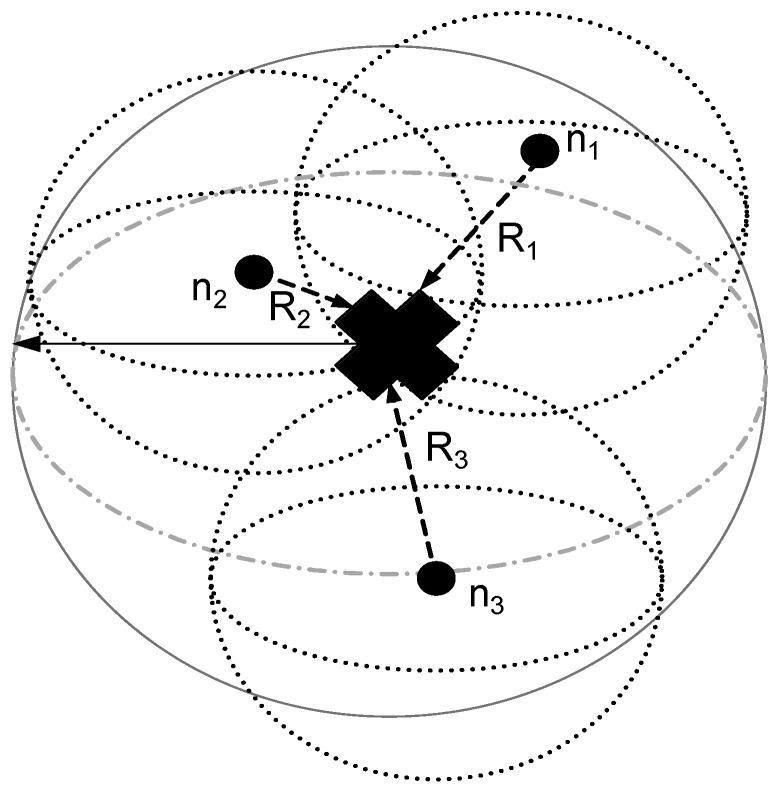
The MS is the neighbor of $n_{i}$ nodes.

If the node $n_{i}$ is in the transmission radius of the MS, this means that $n_{i}$ is the neighbor of the MS, and the data transmission is direct between them, as shown in Figure 5. When the MS moves to the next sink stop, it again finds the neighbor nodes by broadcasting a control packet that contains its location information. $n_{i}$ is a non-neighbor of the MS if it is not in the same RC, where the MS is deployed. Then, it may be a neighbor of any $C_{i}$. Where $C_{i}$ belongs to CNs that move in each of the remaining linear RC regions and gather data from $n_{i}^{\prime }$, $C_{i}$ relays the data of $n_{i}^{\prime }$s to the MS.

To support the proposed model, a set of linear programming equations is given as follows:

Objective function:

(1) $$Maximize\;T=\sum_{s}t_{s_{i}}+\sum_{t}t_{t}\;\forall s_{i}\in S$$

(2) $$\text{subject}\;\text{to}:\;h\left(i\right)\leq d\left(n_{i},n_{j}\right)+h\left(j\right)\;\forall i\in N,j=1,2,3,4$$

(3) $$T_{max}\cdot f_{i}\geq t_{s}\geq T_{min}\cdot f_{i}\;\forall i\in N$$

(4) $$\sum_{j=1}^{4}\sum_{i=1}^{n}d\left(n_{i},n_{j}\right)f_{i}\leq l\;\forall i\in N,j$$

(5) $$T\cdot \left(\sum_{j=1}^{4}\sum_{i=1}^{n}p_{ij}\right)\leq E_{i}\;\forall i\in N,j=1,2,3,4$$

(6) $$E_{i}\left(t_{a}\right)\geq E_{\left(min\right)}^{tx}\;\forall i\in N$$

(7) $$f_{ij}\leq f_{ij}^{max}$$

(8) $$t_{s},f_{i}\geq 0\;\forall i\in N$$

In Equation (1), *T* is the NLT, $t_{s}$ defines the time duration that the MS or CN spends on a stop for data gathering, and $t_{t}$ shows the traveling time of the sink between sink stops. Both $t_{s}$ and $t_{t}$ are calculated till the death of the first node. In Equation (1), the first summation accounts for the total number of sink stops, while the second summation accounts for the total number of travels between the sink stops. $t_{s_{j}}$ is the time duration for the sink to stop at a particular stop, and $t_{t}$ is the traveling time between two stops. Node deployment in the field is random, and the number of nodes on each sink stop may vary. Equation (2) shows the heuristic to find the minimum distance between neighbors and non-neighbors. h(i) is the cost function, and h(j) is a heuristic estimate of the distance from node *i* to node *j*. $d(n_{i},n_{j})$ is the distance between nodes *i* and *j*. In Equation (3), $T_{m}in$ and $T_{m}ax$ represent the minimum and maximum stop time of the sink at a location, respectively. The sink stops at a stop until it receives data from the nodes in its sensing range. $t_{s}$ is the optimal sink stop time period for data gathering. $f_{i}$ is the flow of information (data sensed by nodes). Equation (4) shows that the MS tour will not exceed *l*. The energy conservation constraint is represented in Equation (5), which means that the total energy that nodes consume during the network lifetime cannot exceed the initial energy. When a node *i* transmits data to the nearby sink *j*, it consumes power $p_{ij}$. The transmission of data depends on the residual energy of the node, if it is equal to the minimum energy required for transmission, which is represented in Equation (6). Equation (7) represents the flow constraint through the physical link. It shows that if flow from *i* to *j* exceeds the upper bound $f_{ij}^{max}$, then it results in packet loss/packet drop.

### 4.2. Graphical Analysis

In our proposed scenario, there are two paths for data transmission from nodes to the MS. The first is direct transmission from the node to the MS and via CN. End-to-end delay is greater when the MS receives data from the node via the CN. End-to-end delay is minimum when nodes send data through direct transmission. The total end-to-end delay, denoted by *D*, is the combined delay of direct (D-MS) and multi-hop transmission (D-CN). We define an objective function:

(9) MinimizeD

(10) subjectto:D=(D-MS)+(D-CN)

(11) 87.7≤(D-MS)+(D-CN)≤172.8

(12) 0≤(D-MS)≤87.7

(13) 0≤(D-CN)≤172.8

(14) 0≤(D-MS)+(D-CN)≤260.5

**Figure 6:**
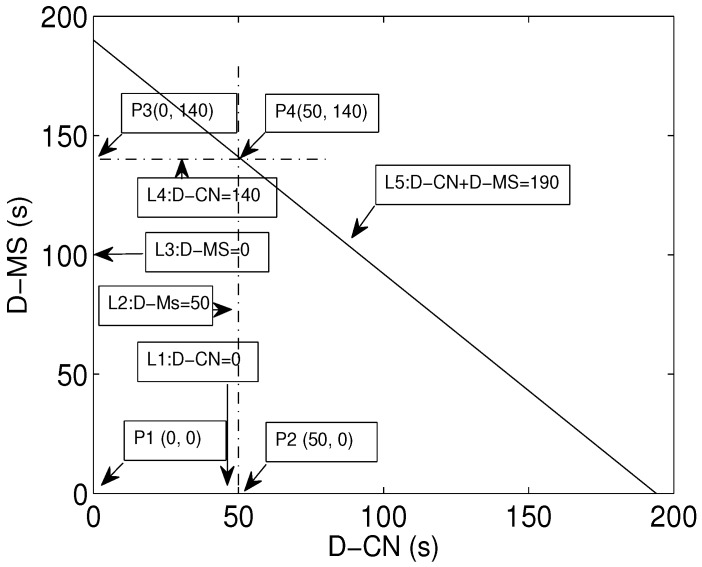
Delay: feasible region.

Equation (9) aims to minimize the end-to-end delay of the network. Equation (10) defines the nature of the delay, *i.e.*, an objective function and a two-dimensional linear programming problem. Constraints in Equation (11) provide lower and upper bounds of the path, respectively. The constraints defined in Equation (12) and Equation (13) are the upper bounds of D-MS and D-CN independently. Equation (14) defines the upper and lower bounds of the network jointly considering both types of delay. Figure 6 shows the set of feasible solutions. There are lines, L1,L2,L3 and L4, intersecting each other, and the region formed by their intersection is the set of all feasible solutions.

The minimum value of each vertex can be obtained as:

at P1(0,0):D=0 s
at P2(87.7,0):D=50 s
at P3(0,172.8):D=140 s
at P4(87.7,172.8):D=190 s

The minimum value of *D* is 0 s, which shows that transmissions are not started yet. It is the initialization phase. The next value of *D* is 50 s. This indicates direct transmission to the MS. Similarly, the next value is 140 s for multi-hop transmission through the CN. The last value is the maximum delay *i.e.*, 190 s; all of values lie within this bound. This shows that the values of delay at each point lie within the boundaries of the illustrated region.

**Figure 7:**
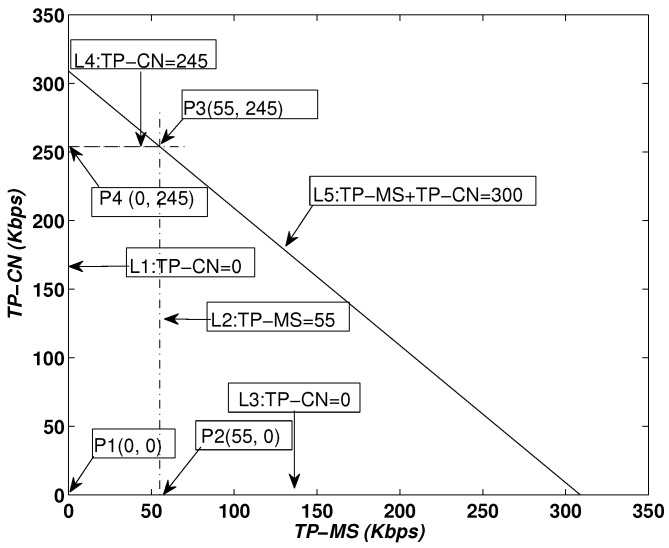
Throughput: feasible region.

During the NLT, the MS and CNs move in the defined RC and collect data periodically, which reduces delays and increases throughput. In Figure 7, the feasible region for throughput is shown; it is represented by units of Kbps. (TP=(TP-MS)+(TP-CN)):

(15) MaximizeTP

(16) subjectto:0≤(TP-MS)+(TP-CN)≤300

(17) 0≤(TP-MS)≤55

(18) 0≤(TP-CN)≤245

The objective function is defined in Equation (15). According to the bounds provided in Equation (16), Equation (17) and Equation (18), Figure 7 shows the intersecting lines L1, L2, L3 and L4, resulting in a bounded region that shows the set of feasible solutions. Values on each vertex are obtain as:

at P1(0,0)=0 Kbps,
at P2(55,0)=55 Kbps,
at P3(55,245)=55+245=300 Kbps,
at P4(0,245)=245 Kbps

The validity of the feasible region is proven. The total throughput during a trip of the MS and CN lies within the boundaries of the illustrated region.

### 4.3. Delivery Probability

In the proposed scheme, we classify nodes into two categories: neighbors of the MS and non-neighbors of the MS. Their delivery probability is given as $del_{prob}=\frac{pkt_{d}}{pkt_{f}}$, where $pkt_{d}$ are the successfully delivered packets by the node and $pkt_{f}$ are the packets sent by the node.

We ignore water currents in our scheme and assume that nodes present in the network are static.

### 4.4. Attenuation and Propagation Delay

Unlike terrestrial WSNs, attenuation in UWSNs does not only depend on the link distance, but also on signaling frequency *f*, *i.e.*, A(d,f), where *A* is the attenuation function. After attenuation, the signal-to-noise ratio (SNR) of the received signal is ρ(d,f) [15]. For distance “*d*” between the source node and the MS (destination) at a frequency *f*(kHz) with spreading coefficient *k*, Urick in [16] defined attenuation equation $A\left(d,f\right)=A_{0}d^{k}a^{d}\left(f\right)$, where $A_{0}$ denotes the normalization constant and a(f) is the absorption coefficient that is described by the Thorp formula [17] and by Equation (19) and Equation (20):

(19) $$10log_{a}\left(f\right)=\frac{0.11f^{2}}{1+f^{2}}+\frac{44f^{2}}{4200+f}+\frac{2.75f^{2}}{10^{4}}+0.003\;if\;f>0.4$$

and:

(20) $$10log_{a}\left(f\right)=0.002+\frac{0.11f}{1+f}+0.011f\;if\;f<0.4$$

This is calculated in dB/km.

The end-to-end delay model in [18] is used to calculate the propagation delay in Equation (21), *i.e.*,

(21) $$T_{p}=\frac{s}{v}$$

where *s* is the distance between the sender and receiver node and *v* is the speed of the acoustic signal, which is given in Equation (22):

(22) $$\begin{array}{cc} v= & 1449.05+45.7t-5.21t^{2}+0.23t^{3}+\\ & \left(1.333-0.126t+0.009t^{2}\right)\left(S-35\right)+16.3z+0.18z^{2} \end{array}$$

where t=T/10, *T* is the temperature in °C, *S* is salinity in ppt, and *z* is the depth in m.

### 4.5. Acoustic Channel Noise

The ocean medium is different than the air medium in WSNs in terms of impedance. In the acoustic channel, the signal is affected by different noises. Primarily, we take into account turbulence $\left(N_{t}\right)$, shipping $\left(N_{s}\right)$, wind $\left(N_{w}\right)$ and thermal noise $\left(N_{th}\right)$. Vakily *et al.* [19] modeled these noises by Gaussian statistics, given as:

(23) $$N\left(f\right)=N_{t}\left(f\right)+N_{s}\left(f\right)+N_{w}\left(f\right)+N_{th}\left(f\right)$$

where,

$$\begin{array}{cc} 10logN_{t}\left(f\right) & =17-30logf\\ 10logN_{s}\left(f\right) & =40+20(s-0.5)+26logf-60log(f+0.03)\\ 10logN_{w}\left(f\right) & =50+7.5\sqrt{w}+20logf-40log\left(f+0.4\right)\\ 10logN_{th}\left(f\right) & =-15+20logf \end{array}$$

Here, *s* is the shipping activity factor, and it ranges 0≤s≤1 and 0≤w≤10 (m/s) in wind velocity.

### 4.6. Energy Consumption Model in UWSNs

Nodes deployed in the underwater network field are powered by batteries. Energy consumed in sensing and processing data is negligible as compared to the data transmission and reception. Transmission power depends on the distance and transmission range. With the propagation model, we estimate the transmit power for a specified signal-to-noise ratio (SNR). We use the energy consumption model for the acoustic communication given in [20]. The SNR equation for passive sonar is calculated in Equation (24):

(24) SNR=SL-TL-NL+DI≥DT

where SL represents the source level, TL is the transmission loss and NL is the noise level of the receiver and the environment. The directive index is denoted by DI. The sonar’s detection threshold is DT. The TL for nodes in the underwater environment is calculated in Equation (25) by the Thorp model in [4], *i.e.*,

(25) $$TL=10log\left(d\right)+\alpha d\times 10^{-3}$$

where *d* denotes the distance between the sender and receiver and *α* is the absorption coefficient. In Equation (24), noise loss is calculated by Equation (23). SL can be calculated from Equation (24):

(26) SL=SNR+TL+NL-DI

Transmitted signal intensity is calculated as:

(27) $$I_{T}=10^{SL/10}\times 0.67\times 10^{-18}$$

and transmitted power by source is:

(28) $$P_{T}\left(d\right)=2\pi \times 1m\times H\times 10^{SL/10}\times 0.67\times 10^{-18}$$

Equation (29) determines the energy required for the transmission of *k* bits over a distance *d*:

(29) $$E_{tx}\left(k,d\right)=P_{T}\left(d\right)\times T_{tx}$$

where $T_{tx}$ is the time in seconds that *k* bits take to reach the destination by covering *d* distance. Consider a network with *N* number of battery-operated wireless nodes; they are randomly deployed in the field, and they will remain stationary afterwards. All of the nodes in the network have an equal sensing range and an equal amount of initial energy. A node performs four major tasks: sensing, computing, reception and transmissions. In UWSNs, nodes use acoustic communication for data transmission, and this has a large propagation delay. To save energy, a sleep/awake mechanism can be introduced into the network. However, due to the long propagation delay, nodes may not be able to receive the control message from the MS and become active immediately. In our proposed scheme, nodes stay active till their death.

UWSNs are different from terrestrial WSNs in terms of the low communication bandwidth and ocean current. This scheme follows the following two steps:

- **Association phase:**
  - The MS has the information of its location $\left(X_{n},Y_{n},Z_{n}\right)$, where *n* = 1, 2, 3, 4.
  - The MS collects data from in range neighbors by calculating the distance, *i.e.*, $d\left(n_{i0}\right)=\sqrt{{\left(x_{i0}-X_{n}\right)}^{2}+{\left(y_{i0}-Y_{n}\right)}^{2}+{\left(z_{i0}-Z_{n}\right)}^{2}}\leq R$, where $n_{i0}$ is a single node with location coordinates $\left(x_{i0},y_{i0},z_{i0}\right)$ and *R* is the radius of the sensing range of the MS.
  - Nodes that are non-neighbors of the MS find the $C_{i}$ for data forwarding. They find minimum distance $C_{i}$, $d\left(n_{i1}\right)=\sqrt{{\left(x_{i1}-x_{ci}\right)}^{2}+{\left(y_{i1}-y_{ci}\right)}^{2}+{\left(z_{i1}-z_{ci}\right)}^{2}}\leq r$, where $n_{i1}$ is the single node with location coordinates $\left(x_{i1},y_{i1},z_{i1}\right)$, *i.e.*, the non-neighbor of the MS, and *r* is the sensing and transmission radius of $C_{i}$.
  - A node sends the sensed data to the MS directly if $d(n_{i})\leq R$; otherwise, it waits until the MS arrives at the nearest stop. The same condition applies for those nodes that send their data to $C_{i}$, if $d(n_{i})\leq r$.
- **Collection phase:** After the start of network operation, all nodes are in active mode and sense data from the field. The MS broadcasts a control packet $I_{c}\left(n_{i},d\left(n_{i}\right),t_{i},R\right)$, where $I_{c}$ is a control packet, $n_{i}$ is the current node ID and $d(n_{i})$ is the distance between the node and MS. $t_{i}$ is the time slot allocated to $n_{i}$ for data transmission, and *R* is the transmission radius of the MS.

Nodes that do not receive the control packet from the MS in a certain time *t* receive a control packet from $C_{i}$, *i.e.*, $i_{c}\left(n_{i1},d\left(n_{i1}\right),t_{i1},r\right)$, where $i_{c}$ is the control packet and $n_{i1}$ is the current node ID pf the neighbor of $C_{i}$. $d(n_{i1})$ is the distance between the nodes and the CN. *r* is the transmission radius of the node.

## 5. Simulation Results

In this section, we evaluate the performance of our proposed 3D-SM with two existing protocols: DDRP-4MS and AUV-PN. The simulation parameters are given in Table 3, where the field dimensions are 500 m × 500 m × 1000 m with 300 nodes that are randomly deployed in the field. The initial energy of each node is 70 J. The transmission radius of the MS is 70 m in each scheme. For a fair comparison, besides the working principles, in three protocols, we consider the same network dimensions, number of nodes, initial energy and the transmission range of nodes. DDRP has multi-hop transmissions for data delivery to an MS, which is collecting data from the nodes and has a random trajectory. In 3D-SM, the MS collects data from nodes directly, and it moves on a straight trajectory in the field, as shown in Figure 3. In those regions where the MS is far away from the nodes, CNs are playing the role of helping to avoid multi-hop communication, and they collect data from the nodes and send them to the MS at the minimum distance. We use the Linprog solver to solve the linear programming model in Section 4.1.

Definitions of the performance parameters are given below:

1. *NLT:* The definition of the network lifetime varies; the authors in [21] state it as the duration till the death of the first node, and the authors in [4] define it as the duration till all nodes die. Similarly, other definitions of network lifetime exist that indicate different percentages of dead nodes while calculating the network lifetime. We stick with the definition of the authors in [21], because in balanced data transmissions, effective communication is only possible till the death of the first node.
2. *Dropped packets:* This is the number of data packets dropped during the transmissions from the node to the sink due to a bad link status. Its unit is the number of packets per second.
3. *Throughput:* The throughput of the network is defined as the number of successfully received packets at the sink. It is also measured as the number of packets per second.
4. *End-to-end delay:* This is defined as the average time taken by a data packet to reach the sink from the source node. Its unit is also seconds.
5. *TL:* This is the accumulative decrease in the intensity of waveform energy as a wave propagates from the source to the destination. In the underwater environment, TL occurs due to attenuation and spreading of the data packet from the source node to the sink. It is measured in dB.
6. *Path loss:* The signal attenuation between a transmit and a sink antenna as a function of the propagation distance is defined as path loss. It is also measured in dB.

### 5.1. NLT

3D-SM balances the load of nodes and results in longer NLT. Exponential node depletion shows that energy consumption of the nodes is balanced, and after the death of the first node, nodes start depleting their energy. This scheme avoids multi-hop transmissions in order to save the energy of nodes. The MS and CNs receive data from the nodes, and then, the CN further transmits the received data to the MS. In terms of NLT, 3D-SM > DDRP > AUV-PN. This analysis shows that AUV-PN consumes more energy in the formation of clusters and in the selection of CHs, which results in a shorter NLT.

**Figure 8:**
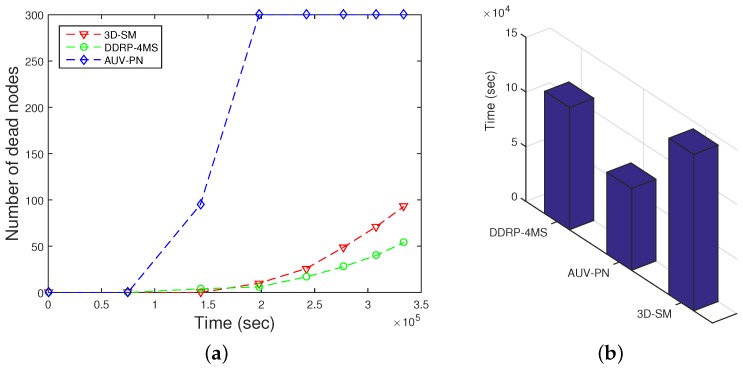
Network lifetime. (**a**) when all nodes die; (**b**) when first node dies.

However, DDRP and AUV-PN both have imbalanced energy consumption, as depicted in Figure 8. Nodes that are selected as CHs have a shorter lifetime because these relay the data of the MN. The selection criteria of the CH is based on the low-energy adaptive clustering hierarchy (LEACH) protocol, which depends on the probability. This is the major flaw of the LEACH protocol; there is no check on the residual energy. In the stable election protocol (SEP) [22], the authors consider a two-level heterogeneous system, where nodes have two different levels of energy: normal and advanced. Advanced nodes are 10% of the total number of nodes in the network, and they have double the initial energy as compared to the normal nodes. In the distributed energy-efficient clustering algorithm (DEEC) [23], the authors consider a multi-level heterogeneous network with normal, advanced, super and ultra-super nodes. In both schemes, CHs are selected on the basis of the maximum residual energy; while in DDRP, there is no set pattern of the MS trajectory such that it will cover the whole network. Its mobility is random, and it may visit the same region twice in its data collection tour; this leads to imbalanced transmissions, as shown in Figure 8.

### 5.2. Throughput

**Figure 9:**
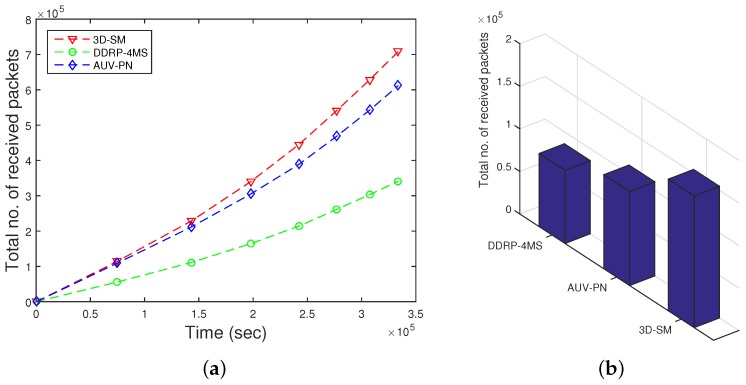
Network throughput. (**a**) when all nodes die; (**b**) when first node dies.

The amount of data sent to the sink depends on the number of nodes that are alive and participating in sensing data. 3D-SM is designed in such a way that the MS efficiently covers the areas and gathers data from nodes at the least distance. Nodes send their data to the MS or CNs directly, which results in a network with higher throughput. In Figure 9, throughput is shown; 3D-SM performs better due to the longer NLT and the mobility pattern of the MS. CNs frequently receive data from the nodes and send these data to the MS; this results in greater throughput. Figure 9 shows that initially, both schemes, 3DSM and AUV-PN, have almost the same throughput, but with the passage of time, 3D-SM takes the lead. The energy depletion of nodes directly affects the throughput of the network. In AUV-PN, when the stability period is over, the throughput also decreases, due to the decrease in the number of nodes present in the network.

In DDRP, when the MS starts the data gathering tour, it stays at different sink stops. When it stays at the first stop, it broadcasts a control packet that has information about its stop location, sensing range, time of stay, *etc*. The nodes that are present in the sensing radius of the MS attach themselves to the MS. The nodes that do not receive a control packet broadcast a message to find the route towards the MS. The nodes that are a one-hop neighbor of the MS send their data directly. The nodes that are two or more hops away send data packets through multi-hop communication. In multi-hop communication, the chances for packet drops increases, which leads to low throughput. In this scheme, an MS gathers data from the whole network and randomly moves in the field. Due to the random mobility, the MS may not receive all of the information from the field. DDRP achieves higher NLT as compared to AUV-PN, however, at the cost of throughput. Initially, the behavior of the plot is linear, as in all three protocols, all of the nodes are alive.

### 5.3. Packet Dropped

**Figure 10:**
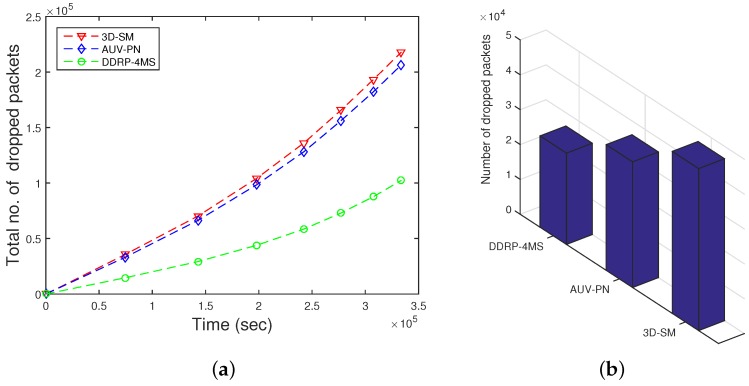
Dropped packets. (**a**) when all nodes die; (**b**) when first node dies.

When the number of transmissions and receptions increases, the packet drop ratio also increases due to collisions. In the simulation, we used the random uniform model [24], where the packet drop is related to the bad link status through which it is propagated. If the link status is good, the sink successfully receives a data packet; on the other hand, if the link status is bad, the packet is dropped. In our simulations, we set a 30% probability of a bad link status, which means $p_{bad}=0.3$ and $p_{good}=0.7$. CNs gather data and deliver them to the MS; when these have more data to deliver in a limited time, then the packet drop ratio increases. Dense networks have higher packet drop ratios as compared to sparse ones. In Figure 10, the packet drop ratio of 3D-SM is greater than DDRP because it has more packets to transmit to the sink, so the probability of packet dropping also increases.

In AUV-PN, there is a greater probability of packet drops, as this protocol engages two-hop transmission of the MN’s data through the CH to the MS. Furthermore, it depends on the number of packets sent to the sink; DDRP’s throughput is less in comparison with 3D-SM.

### 5.4. Path Loss

During the transmissions in the acoustic model, energy loss occurs along the propagation paths. The primary mechanisms for the energy loss are: geometric spreading, absorption loss and scattering loss. Geometric spreading is defined as a sound wave moving away from the node; the area that the sound energy covers becomes larger, and as a result, the sound intensity decreases. The underwater medium for propagation is dense as compared to the terrestrial one, so when an acoustic signal propagates from the source node towards the MS and the distance between them is great, the power loss is caused by the geometric spreading, which is directly proportional to the square of the distance. When the source node transmits the signal, it may be converted to other forms and absorbed by the medium. In the case of acoustic signals, the absorption coefficient is calculated in [10]. Scattering is a physical process where non-uniformities in the medium, like particles and bubbles, force the signal to deviate from its trajectory. Due to the dense underwater channels, there are more chances of scattering. Scattering causes path loss, as well as end-to-end delay.

**Figure 11:**
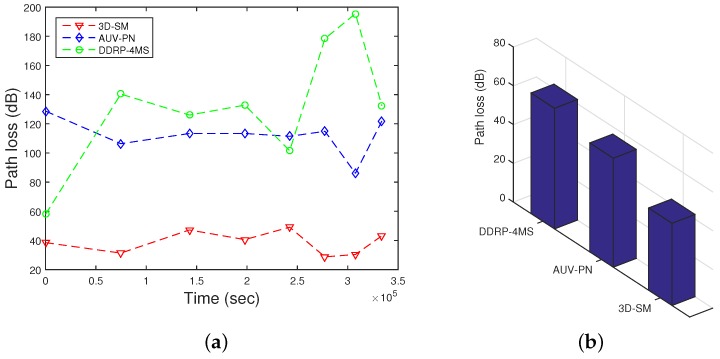
Path loss. (**a**) when all nodes die; (**b**) when first node dies.

In Figure 11, path losses are shown. In 3D-SM, path loss is less in comparison. The whole plot of 3D-SM does not change as drastically as AUV-PN and DDRP. AUV-PN is a cluster based with MS routing protocol; path loss linearly increases at a higher rate in comparison, up till the NLT. After that, around 70,000 s, its behavior slightly varies; however, when the number of dead nodes is increased, the path loss also increases because the network becomes sparse. In DDRP, during the NLT, there is a slight increase in the path loss; however, after the death of the first node, the path loss decreases, because nodes start dying, which leads to an increasing time and abrupt change in path loss when the network is sparse. In 3D-SM, the NLT is longer compared to the existing schemes due to balanced transmissions between nodes and the MS (or CNs). The network is designed in such a way that nodes transmit the sensed data when the distance from the MS (or CN) is the least and avoid path loss.

### 5.5. TL

TL is defined as the decrease of the signal intensity through the path from the sender node to the receiver node. Diverse empirical expressions to measure the TL have been developed. The Thorp formula defines the signal TL (Equation (25)).

**Figure 12:**
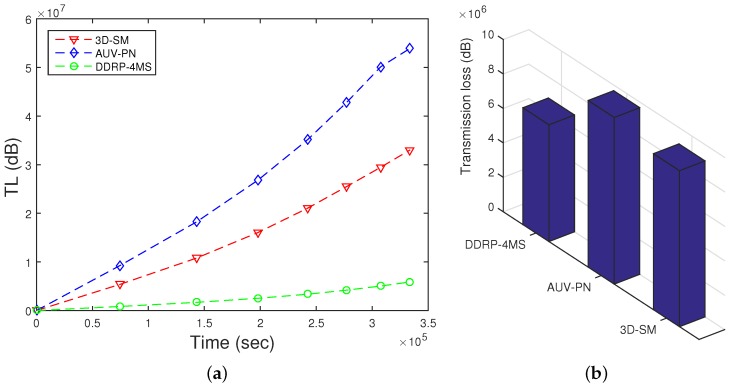
TL of network. (**a**) when all nodes die; (**b**) when first node dies.

TL for the proposed scheme is shown in Figure 12. The results show that the TL of 3D-SM is greater than DDRP. In 3D-SM, the number of transmission is greater, so the TL depends on the distance between the sender and receiver. In this scheme, the transmissions are direct, and distance varies from the minimum value to the maximum (transmission range). The nodes far from the MS also directly send data, which increases the TL in the scheme. In DDRP, the data from the source to the receiver are sent through multi-hop transmission at the minimum distance. This decreases the TL.

The TL of AUV-PN is higher due to clustering. This is the cost paid to achieve better throughput and less delay.

### 5.6. End-to-End Delay

**Figure 13:**
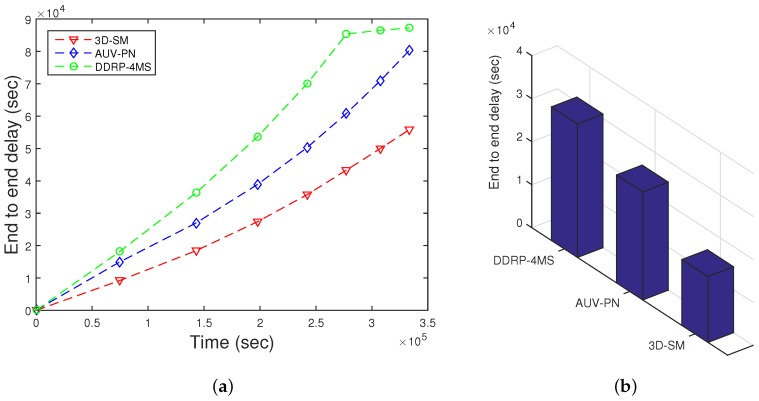
End-to-end delay. (**a**) when all nodes die; (**b**) when first node dies.

End to end delay depends on the speed of the acoustic signal and the transmission distance. Here, *s* denotes the distance between the source node to the MS/CN; the speed of acoustic signal varies from 1450 m/s to 1500 m/s, depending on the depth of the water. As t=s/v, end-to-end delay is directly proportional to the communication distance. In Figure 13, end-to-end delay of three schemes is presented. On the x-axis, the lifetime of the network is shown. On the y-axis, cumulative values are plotted. DDRP has greater end-to-end delay because nodes wait until the MS arrives at a nearby stop to receive data; otherwise, data are forwarded via multi-hopping. In AUV-PN, nodes transmit the sensed data to their respective PN’s. When the AUV starts the data gathering tour, first, it obtains the list of PNs from CHs, and afterwards, it visits each PN for data gathering according to the list. This procedure increases the delay. In our proposed scheme, 3D-SM, the MS gathers data form the nodes after a regular interval. In the rest of the regions, CNs transmit the collected data to the MS, which leads to relatively lower end-to-end delay. After the stability period ends, nodes start to drain their energy. Once a node drains its energy, it is no longer able to participate in sensing and transmission; we call it a dead node. As the number of dead nodes increases in the network, the sparsity also increases, which leads to end-to-end delay. Interestingly, the end-to-end delay seems inappropriate for delay-tolerant applications. However, our solution is appropriate, as the calculated value (140,000) of end-to-end delay is cumulative; its average value is 190 s.

## 6. Scalability Analysis

Our proposed 3D-SM and the existing AUV-PN and DDRP are simulated by varying the number of nodes in the network ranging from 200 to 1000.

**Figure 14:**
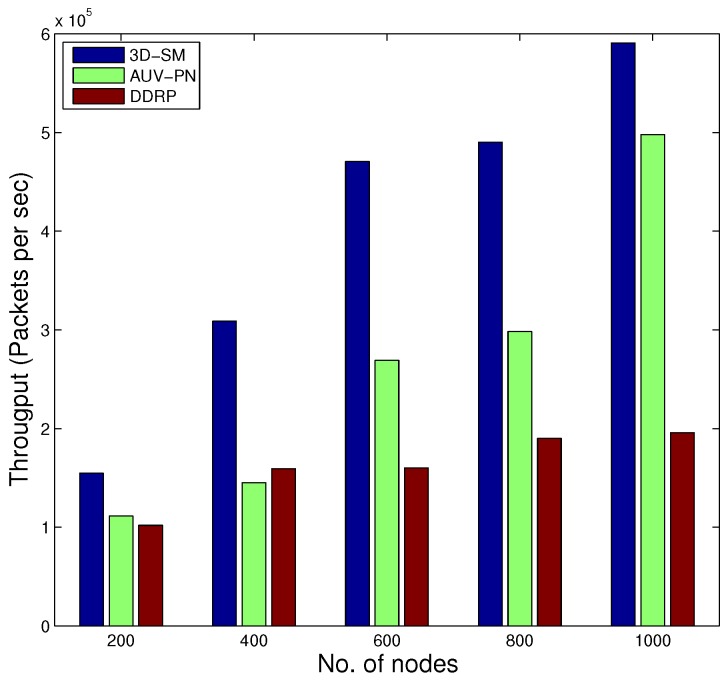
Throughput comparison with different numbers of nodes.

**Figure 15:**
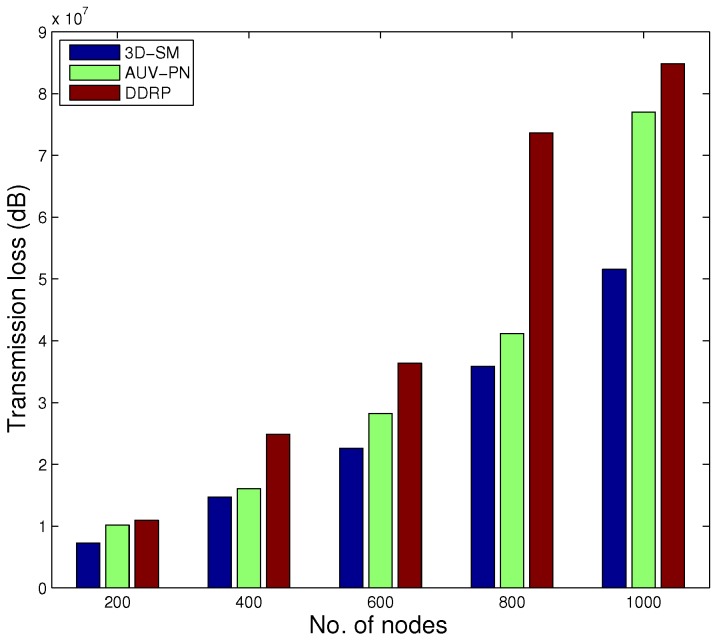
Transmission loss.

**Figure 16:**
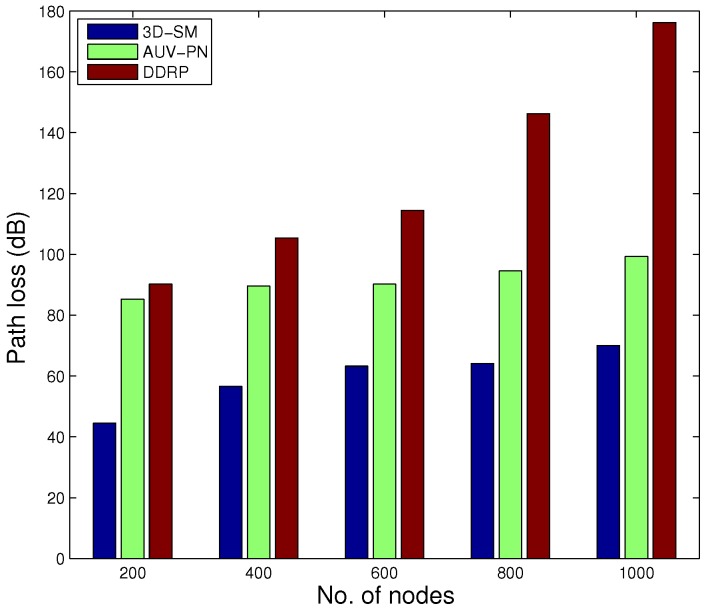
Path loss.

**Figure 17:**
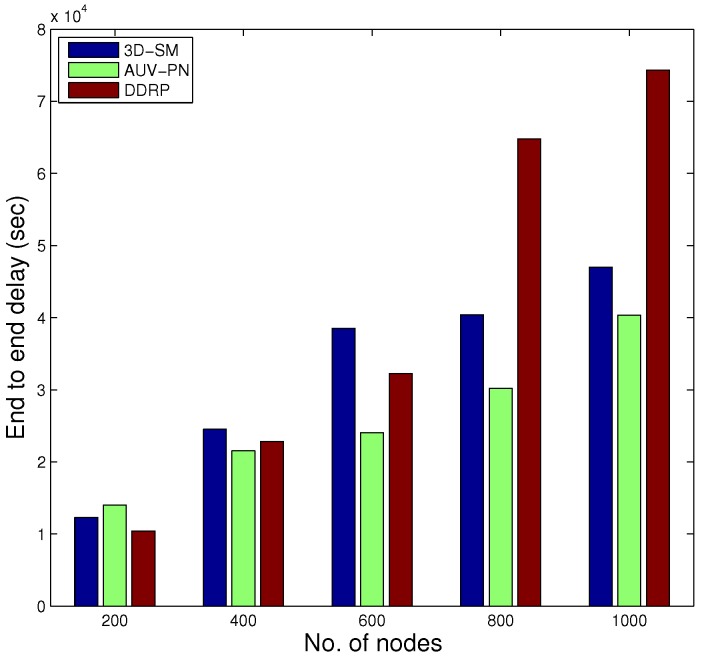
End-to-end delay.

In Figure 14, the behavior of the throughput of the three routing protocols with different numbers of nodes is shown. By increasing the nodes in the network with the same dimensions for SM-3D and AUV-PN, throughput has an increasing behavior; while in DDRP, there is a slight change in the throughput. DDRP supports multi-hopping; with the increase in the number of nodes, the maximum nodes send their data through multi-hoping, which increases the transmission losses (Figure 15), path losses (Figure 16) and end-to-end delay (Figure 17). While AUV-PN and SM-3D are receiving data directly from nodes, end-to-end delay may increase relatively.

Figure 15 shows the transmission losses, with the change in the number of nodes. In SM-3D, transmission losses linearly increase. In AUV-PN and DDRP, transmission losses exponentially increase with increased node density.

Path loss with increased node number is shown in Figure 16. There is a slight variation in the path loss value in 3D-SM and AUV-PN, while in DDRP, by increasing the number of nodes in the network, the path loss also increases exponentially.

As the node density in the network increases, the end-to-end delay of 3D-SM increases as compared to AUV-PN. In AUV-PN, more clusters are formed, and also, the number of PNs increases, as well as the load on the PNs is managed in terms of end-to-end delay. While in 3D-SM, the number of the MS and CNs is fixed, by increasing the number of nodes, the MS and CNs get burdened, and the delay increases.

In DDRP, end-to-end delay exponentially increases because of multi-hopping.

**Figure 18:**
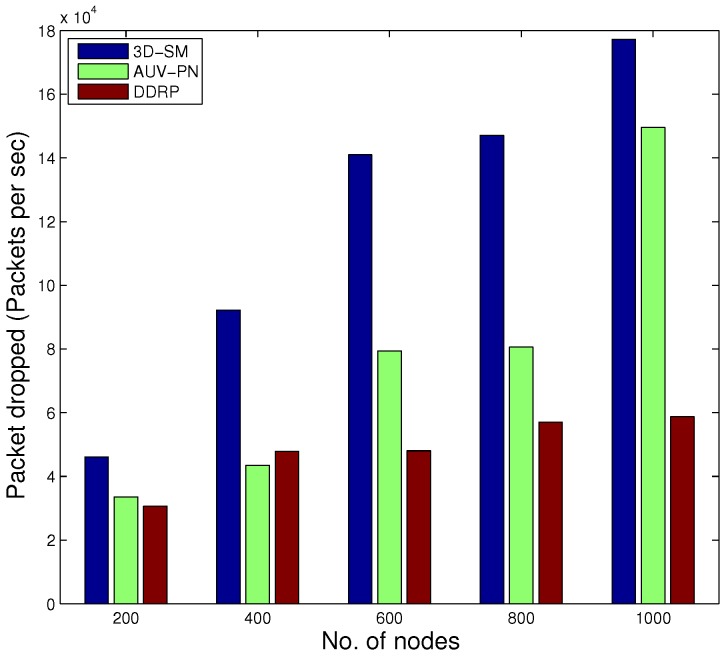
Packet drop ratio.

Figure 18 shows that 3D-SM achieves high throughput at the cost of increased packet drop.

We concluded from this analysis that 3D-SM is more scalable as compared to AUV-PN and DDRP.

## 7. Fixed Routing and Dynamic Sink Mobility

**Figure 19:**
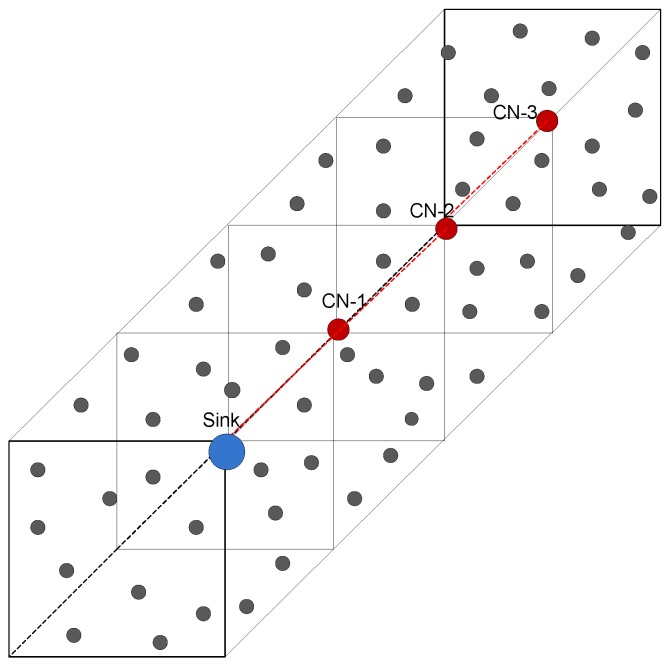
Architecture for fixed routing.

In our proposed scheme 3D-SM, we consider sink mobility to enhance the performance of the network. We consider a network field with dimensions 500 m × 500 m × 1000 m. The network is logically divided into four RCs. In each RC, either the MS or CN is deployed for data gathering from nodes at the least distance. The CN gathers data from its RC and further sends it to the MS. In order to find the difference between fixed routing and sink mobility, we perform simulations, where the network dimensions and number of CNs and MS are kept the same for both fixed routing in 3D-SM. In Figure 19, the architecture of fixed routing is shown. The sink and CN are static, and they are receiving data through direct communication (in order to keep the comparison fair). Far away CNs send their data through multi-hop communication, shown in Figure 19.

**Figure 20:**
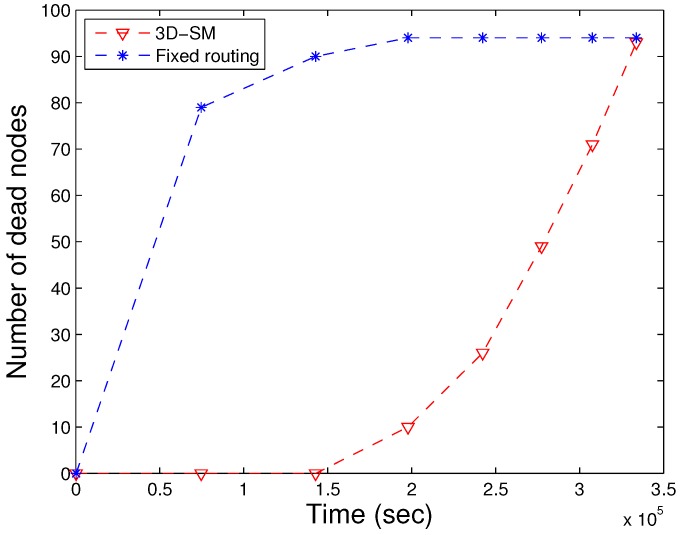
NLT of fixed and dynamic routing.

We compare the results of both schemes in the same environment; one is fixed routing, and the other is the proposed scheme, 3D-SM. As we defined NLT as the time duration from the start of the network till the death of the first node, in Figure 20, the NLT of 3D fixed routing is less compared to 3D-SM, because of the static sink and CNs. The load between the nodes is managed in 3D-SM, which prolongs the network lifetime. In fixed routing, the distance between nodes and the sink (or CNs) is fixed, so the nodes that are far drain their energy quickly.

**Figure 21:**
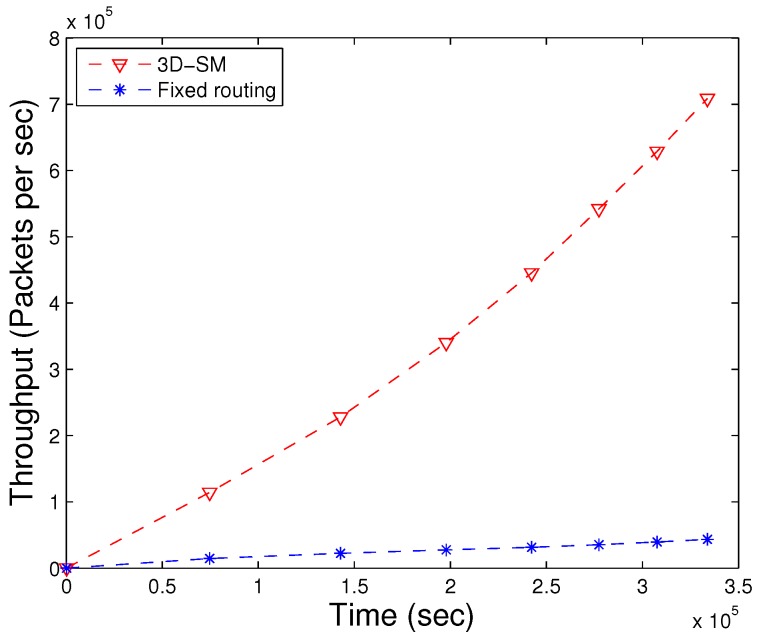
Throughput of fixed and dynamic routing.

**Figure 22:**
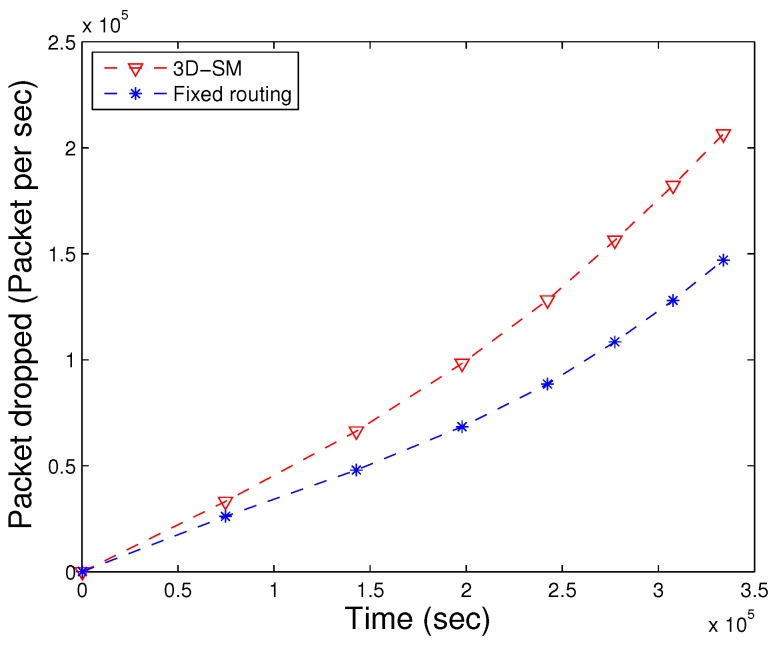
Packet drops of fixed and dynamic routing.

The MS and CNs, when moving in the network, actively gather data from the nodes in 3D-SM. However, nodes are sending the data through fixed routes to CNs (or the sink), and the throughput of fixed routing is less compared to 3D-SM, as shown in Figure 21. In Figure 22, the packet drop of both schemes are shown. In fixed routing, the ratio of the packet drops is high compared to the throughput (the number of packets successfully delivered). Because of the distance and underwater harsh environment, nodes that are far from the sink (or CNs) lose the transmitted packet. The packet dropped ratio also increases when CNs forward the received data through multi-hop communication.

**Figure 23:**
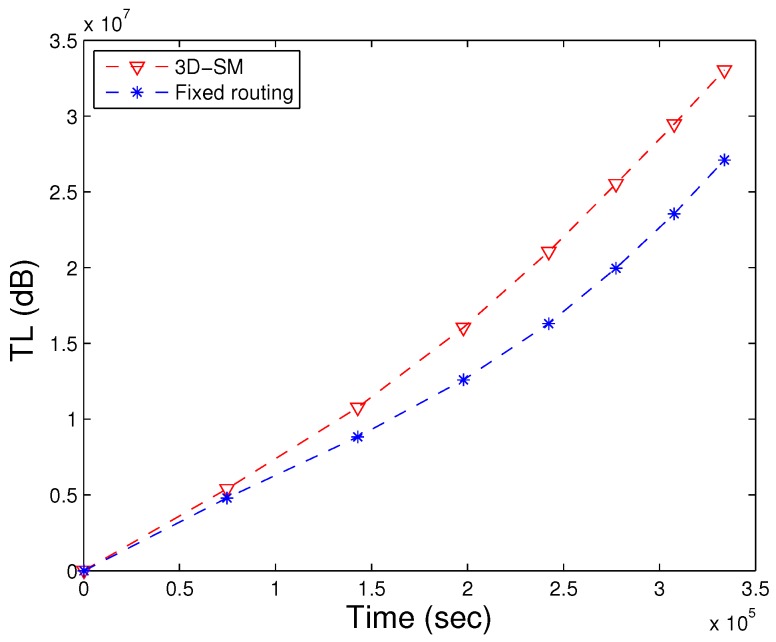
Transmission loss of fixed and dynamic routing.

**Figure 24:**
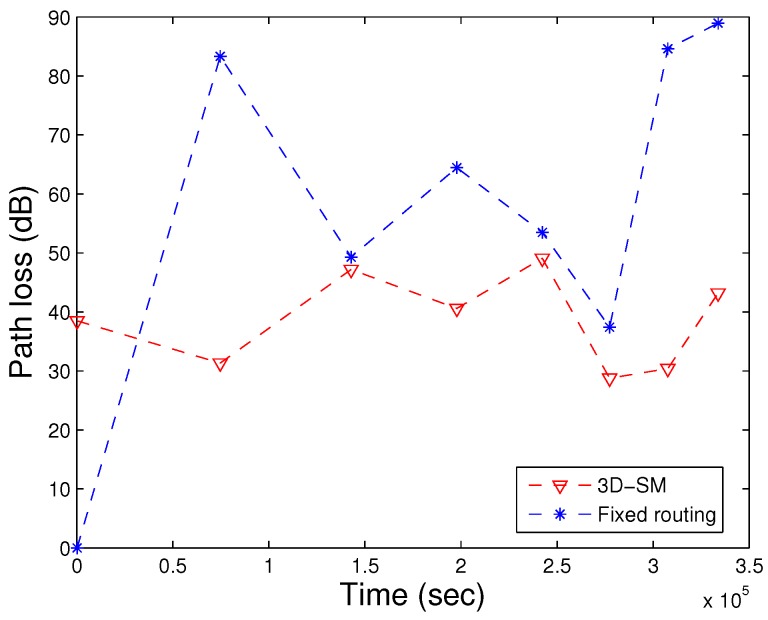
Path loss of fixed and dynamic routing.

**Figure 25:**
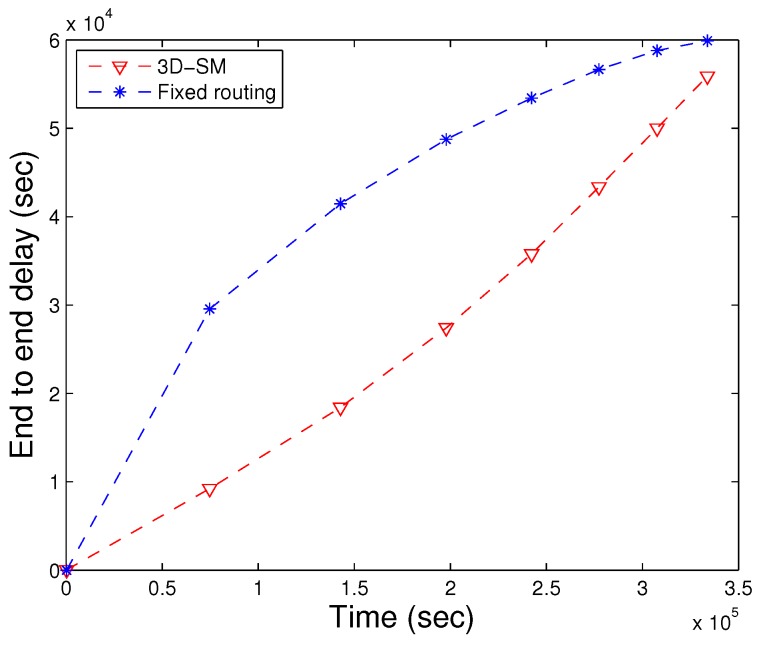
End-to-end delay of fixed and dynamic routing.

In Figure 23, 3D-SM has higher transmission losses compared to fixed routing, because when the network operation starts, nodes quickly die in fixed routing. With the decrease in the number of alive nodes, the transmission loss also decreases. In Figure 24, the path loss of both schemes is represented. Due to the far distance transmission, path losses increase in fixed routing. Nodes send their data directly to the MS (or sink) in both schemes, 3D-SM and fixed routing. In fixed routing, nodes and CNs (sink) are fixed, and far away nodes consume more energy during data transmission; also, the delay increases, as shown in Figure 25.

## 8. Trade-Offs Made by the Protocols

The trade-offs made by the selected routing protocols are given in Table 4 3D-SM has increased throughput, however, at the cost of increased packet drops. The transmission pattern here is direct; after sensing the data, nodes wait for the MS or CN to send them data. In this way, end-to-end delay is minimized by introducing an efficient tour of the MS, as well as CNs. The presence of the CN is contributing to the NLT maximization; however, at the same time, this is the reason for the TL in 3D-SM. We applied a random uniform model for calculating the packet drops, where the packet drops are directly proportional to the throughput; the higher the throughput, the higher the packet drop ratio.

DDRP minimizes the TL and path loss at the cost of reduced throughput, increased end-to-end delay and reduced NLT. DDRP has long end-to-end delay due to the random trajectories of the MS. Furthermore, there is no restriction that the MS will gather data from all nodes in a tour.

AUV-PN is a cluster-based routing protocol in which the PN receives data from the MN and sends them to the AUV. Its NLT is less as compared to the rest of the schemes, because the nodes consume surplus energy in the clustering process. AUV-PN minimizes delay and prolongs NLT at the cost of TL and path loss.

## 9. Conclusions

In this paper, we have proposed an efficient routing protocol, *i.e.*, 3D-SM for UWSNs. It is implemented on a linear network. To minimize the energy consumption of nodes, the whole field is logically divided into RCs, and also, the MS and CNs are deployed. The mobility of the MS and CNs increases the NLT and also minimizes delays. In order to validate our scheme, we compared it to selected existing schemes. Simulation results show that 3D-SM performs better than DDRP underwater and AUV-PN in terms of NLT, throughput, delay, path loss and scalability. Furthermore, we presented a scalability analysis that shows that 3D-SM is scalable. In the future, we will vary the network dimension and also vary the number of MSs and CNs for presenting a realistic approach towards scalability.

## Figures and Tables

**Table 1:** Characteristic differences between terrestrial wireless sensor networks (TWSN) and underwater wireless sensor networks (UWSNs).

Feature	Terrestrial	Underwater
Signal	Radio	Acoustic
Speed	Light speed (3$\times 10^{8}$ m/s)	Acoustic signal speed 1500 m/s
Anchor	GPS-based	AUV
Signal bandwidth	High	Low
Location error rate	Low	High
Device mobility	Static and mobile	Static and mobile
Propagation delay	Low	High
Power source	Battery, solar	Battery

**Table 2:** Overview of existing protocols. AURP, AUV-aided underwater routing protocol; PN, path node; MN, member node; NLT, network lifetime; DFR, directional flooding-based routing.

Scheme	Features	Performance Achieved	Cost Paid
**AURP** [3]	Heterogeneous acoustic channel where multiple AUVs are gathering data from nodes through gateway nodes.	Maximized delivery ratio, minimized energy consumption.	Increased delay.
**Data collection** [5]	The AUV collects data from the nodes. The network is logically divided into four sub-regions, and each region selects a CH. Each sub-region is further divided into clusters, and each cluster selects a PN, which collects the data from MNs and forwards these to the AUV.	Minimized total energy consumption, maximized throughput and minimized overhead.	End-to-end delay increases.
**Delay-sensitive routing schemes for underwater acoustic sensor networks** [7]	Depth-based localization-free routing. CNs gather data and forward these to the surface sink.	Minimized total energy consumption, minimized average end-to-end delay and minimized transmission loss.	Decreased throughput.
**Energy efficient depth based routing (EEDBR)** [6]	Depth-based localization-free routing. Energy consumption is balanced. Sender-based approach, where the sender selects a limited number of suitable forwarding nodes.	Extended NLT, minimized energy consumption and minimized end-to-end delay.	Decreased delivery ratio.
**DFR** [8]	Nodes are location and neighbor aware. Replaces the forwarder in the case of a weak link. Forwarding activity is performed hop by hop. Delivers packets through flooding.	Increased packet delivery ratio and less communication overhead.	Increased delay.
**Mobicast** [9]	Nodes’ positions can be changed due to water currents. The AUV moves on a user-defined trajectory and collects data from 3D zones. Nodes record their location in a specific time period to calculate the drift and also observe the sleep/awake mechanism. Capable of covering the drift distance of a node.	Minimized delay, increased throughput and increased successful delivery rate.	Increased message overhead and increased power consumption.
**Adaptive surface sink replacement strategy** [10]	The surface sink is capable of self-configuration and has no energy constraint. The sink updates the table of node IDs and their energies, and the next time, when receiving data, it compares to the existing record either the same node or it changes. If the nodes’ power level is changed, then the sink will change its position in the (x,y) plane to reduce the distance between nodes to minimize the energy consumption.	Minimizes energy consumption and end-to-end delay.	Decreased throughput.
**Remote data retrieval strategy** [11]	Head nodes receive data from the neighbor nodes and forward these to the AUV in a grid topology.	Increased packet delivery ratio, decreased end-to-end delay and minimized energy consumption.	Increased end-to-end delay.

**Table 3:** Simulation parameters.

Operations	Values
Initial energy of node	70 J
Transmitter electronics	6.3 mJ/bit
Data rate	1 Kbps
Transmission range	70 m
Network depth	500 m
Network breadth	1000 m
Network width	500 m
Total number of nodes	300
AUV	1
CNs	3

**Table 4:** Performance trade-offs made by the protocols. DDRP, data-driven routing protocol.

Protocols	Achieved Parameters	Reference	Compromised Parameters
**3D-SM**	NLT extended	Figure 8	Increases TL (Figure 12) due to CNs and also increases the packet dropped (Figure 10) ratio, as it is 0.3% of the throughput.
**DDRP**	TL is reduced	Figure 12	Increased end-to-end delay (Figure 13) due to direct transmission of data from the node to the MS and also the random path of the MS.
**AUV-PN**	Minimizes end-to-end delay	Figure 13	TL increases due to multi-hop transmissions (Figure 12). Decreased NLT (Figure 8) and increased path loss (Figure 11)
**3D-SM**	Improves throughput	Figure 9	At the cost of TL (Figure 12).
**DDRP**	Minimizes path loss	Figure 11	Due to the random mobility of the MS, and direct data transmission throughput decreases (Figure 9)
**AUV-PN**	Improves throughput	Figure 9	Path loss (Figure 11) and TL (Figure 12) increase due to multi-hoping and clustering.
**3D-SM**	Minimizes end-to-end delay	Figure 13	Increased packet dropped ratio (Figure 10) due to increased throughput (Figure 9).
**3D-SM**	Minimizes path loss	Figure 11	Due to the high throughput ratio, the packet dropped ratio increases (Figure 10).
**DDRP**	Minimizes path loss	Figure 11	Decreased throughput (Figure 9) and increased end-to-end delay (Figure 13)
